# Bottom-up supramolecular assembly in two dimensions

**DOI:** 10.1039/d1sc05667k

**Published:** 2022-01-19

**Authors:** Ignacio Insua, Julian Bergueiro, Alejandro Méndez-Ardoy, Irene Lostalé-Seijo, Javier Montenegro

**Affiliations:** Centro Singular de Investigación en Química Biolóxica e Materiais Moleculares (CiQUS), Departamento de Química Orgánica, Universidade de Santiago de Compostela 15705 Spain javier.montenegro@usc.es

## Abstract

The self-assembly of molecules in two dimensions (2D) is gathering attention from all disciplines across the chemical sciences. Attracted by the interesting properties of two-dimensional inorganic analogues, monomers of different chemical natures are being explored for the assembly of dynamic 2D systems. Although many important discoveries have been already achieved, great challenges are still to be addressed in this field. Hierarchical multicomponent assembly, directional non-covalent growth and internal structural control are a just a few of the examples that will be discussed in this perspective about the exciting present and the bright future of two-dimensional supramolecular assemblies.

## Abstract 2

The self-assembly of molecules in two dimensions (2D) is gathering attention from all disciplines across the chemical sciences. This perspective discusses the main strategies to direct the supramolecular self-assembly of organic monomers in 2D.

## Graphical abstract

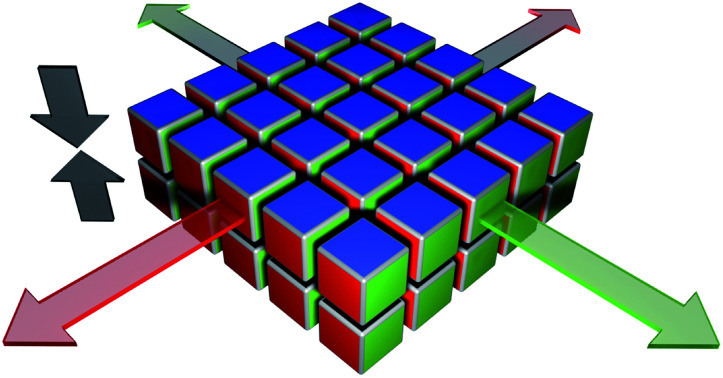

## 1. Introduction

The discovery of graphene in 2004 sparked interest in 2D materials with broad cross-disciplinary resonance.^1^ In the two following decades, other covalent 2D architectures^2^ and inorganic allotropes were developed, such as elemental 2D films^3^ and metal–organic frameworks,^4^ amongst others.^5^ The intriguing properties of different 2D molecular systems are currently attracting great attention towards tackling important societal challenges such as green energy sourcing,^6^ new-generation catalysts^7^ and biomedical technologies.^8^ Alternatively to static covalent and inorganic nanomaterials, supramolecular 2D assemblies allow controlled structural and functional reconfiguration.^9^ The design of small self-assembling monomers bearing orthogonal and directional binding motifs has led to a plethora of supramolecular 2D nanomaterials.^8,10^ The non-covalent foundation of these systems allows the dynamic exchange of monomers and structural remodelling in response to external conditions (*e.g.* temperature, pH, and ionic strength),^11^ which endows the resulting materials with new functional properties such as self-healing,^12^ polymorphism,^13^ or molecular capture and sensing.^14–16^ Despite the continuous development of new supramolecular 2D materials, there is still a know-how gap in predicting the supramolecular outcome of monomers at the molecular design stage. The establishment of precise assembly guidelines remains elusive, so each new monomer must undergo its own structural optimisation. Since a single monomer can in many cases self-organise into several supramolecular ensembles, many efforts currently focus on controlling the assembly pathway towards isolating a single supramolecular product (see Section 2).^17,18^ To control competing assembly pathways, external conditions such as heating–cooling ramps, solvent exchange or time-dependent structural interconversion must be screened.^19^

**Fig. 1:**
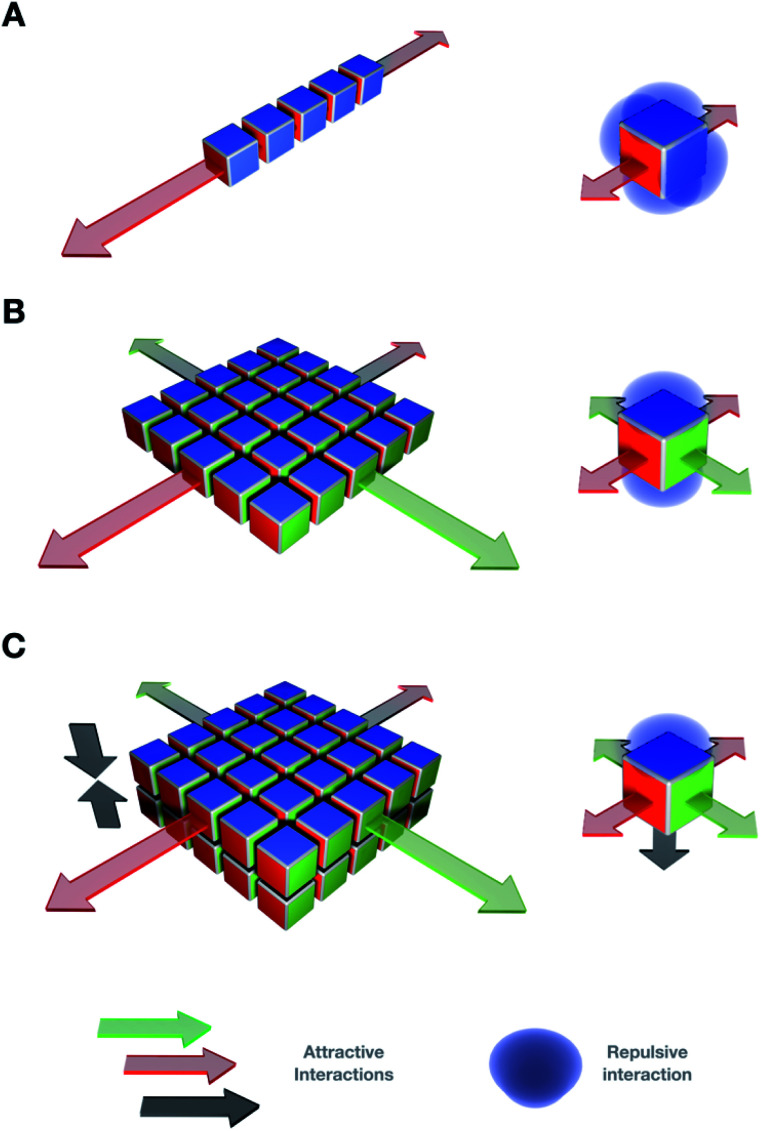
General design strategy to direct monomer self-assembly in 2D. The orientation of several attractive interactions, both directional (*e.g.* H-bonding, π–π stacking, *etc.*) and non-directional (*e.g.* hydrophobic effects), on synthetic monomers – depicted here as cubes – determines the supramolecular propagation in the different dimensions of space. (A) One single attractive interaction (red) can be used to achieve one-dimensional elongation. (B) Two orthogonal interactions (red and green) in perpendicular orientation can propagate the 2D assembly. (C) Incorporation of a third attractive interaction (grey) allows for 2D bilayers to form. In all cases, repulsive interactions (*e.g.* electrostatic, in blue) prevent further aggregation of the assembly.

Simple molecules with few non-covalent binding domains, which would not be localised or directionally restricted, can display high rotational freedom and different potential assembly pathways. However, under specific external conditions (*e.g.* physiological), the highest degree of structural accuracy and assembly reproducibility is achieved by complex monomers with a high number of precisely positioned non-covalent binding points (*e.g.* tubulin). In this framework, a two-dimensional assembly is achieved by controlling the orientation of non-covalent interactions on the suitable synthetic monomers (Fig. 1), whereas high internal order would be required to control material properties such as surface functionalisation and morphology; static crystalline assemblies display limited stimuli-responsiveness due to a high structural stability. Hence, a key challenge towards new-generation 2D supramolecular materials lies in understanding the geometrical constraints required for monomers to assemble highly ordered molecular lattices and avoid competing assembling pathways, while maintaining an adaptive stimuli-responsive behaviour.

In this perspective, we will discuss concepts in supramolecular polymerisation and monomer design distilled from the literature on aqueous 2D assemblies.^8^ The ideas presented here navigate the structure-assembly relationships of monomers, aiming to balance assembly complexity and ordered monomer packing in 2D. Firstly, supramolecular polymerisation strategies will be presented with a focus on controlling polydispersity, aspect ratio and other key structural features of 2D nanomaterials. Secondly, monomer design will be discussed from a geometrical perspective, addressing the orientation of supramolecular interactions to promote internal order in 2D ensembles. Finally, a discrete collection of edge applications in chemistry, materials science and biology is presented to highlight the current and potential uses of these advanced nanomaterials.

## 2. Assembly mechanisms and strategies

Control over the assembly mechanisms is key to direct precise supramolecular architectures with dimensional order. A combination of at least two orthogonal non-covalent forces is required to elongate monomers in 2D (Fig. 1).

However, the inherent complexity of non-covalent interactions still hinders a precise framework for multidimensional assembly prediction.^20,21^ Recent discoveries contribute to the potential prediction of supramolecular multiscale materials by molecular design, both generally and specifically in 2D. Different concepts and mechanisms important for two-dimensional supramolecular polymerisation will be discussed in this section.

### 2.1. Energy landscape

Contrary to covalent systems, supramolecular polymers are frequently under thermodynamic control, particularly under dilute conditions. Nonetheless, supramolecular assemblies have also shown kinetic control in solution.^22^ This scenario evolves into the definition of *pathway complexity*, which addresses the variety of supramolecular products that can originate in solution from a single monomer.^18^ Small changes in the monomer structure can produce radical changes in the supra-polymerisation mechanism, transiting from thermodynamic to kinetic control and even generating different metastable states.^23^ Even small amounts of co-solvent in the ppm range can also alter the system's energy and the expected assembly pathway.^24^ Solution properties such as ionic strength may also impact the shape and internal order of the assembly.^25^ Environmental factors such as mechanical stress produced at moving air–water interfaces can play an important role in the energetic assembly pathway.^13^ The control of the assembly pathway can also have a drastic effect on the size and number of defects of supramolecular 2D assemblies.^26,27^ Therefore, all factors affecting the assembly pathway have to be considered when designing a potential monomer for 2D self-assembly.^28^

### 2.2. Suprapolymerisation mechanism

Two main general mechanisms can be considered: *isodesmic*, where monomer association constants do not vary along the polymerisation process (such as covalent step-growth polymerisation), and *cooperative* (chain-growth), in which monomer association constants vary with polymer length.^29^ First reports of living chain-growth supramolecular polymerisation gave hints on the basic molecular design principles needed to achieve control over block copolymerisation.^30^ This strategy also allowed a certain degree of control in the suprapolymer's length and molecular weight.^31,32^ Monomer design has been employed to control the chain-growth polymerisation mechanism to afford bidimensional materials.^33^ For generating 2D structures, it could be desirable to ensure higher polymerisation degrees in the sheet's plane. Here, a cooperative mechanism would ensure that the polymer weight fraction is skewed towards a higher degree of polymerisation.^29^ However, the precise translation of the mechanistic aspects of supramolecular polymerisation for the construction of 2D assemblies is not always a trivial task.

### 2.3. Supramolecular copolymerisation

The combined polymerisation of different supramolecular monomers allows access to hybrid materials with new assembly capacities and functionalities.^21^ However, the dynamics of non-covalent forces can hinder the control of the internal microstructure of supramolecular copolymers compared to covalent analogues.^34^ Block and alternating isodesmic supramolecular copolymerisations have been achieved, mostly for 1D assemblies, taking advantage of the directional interaction of H-bonding donor–acceptor groups.^35^ Temperature-controlled copolymerisations were reported to be assembled into block tubular^36^ and fibrillar^37^ nanostructures. Specific host–guest interactions can also be employed in dynamic self-sorting of sequence-controlled 1D polymers.^38^ Fluorescently labelled monomers have recently allowed the structural characterisation of multiblock tubular polymers with low polydispersity.^39,40^ More challenging is block segregation in 2D, which has been achieved by thermodynamic assembly of flexible monomers into lamellar phase-segregated blocks.^27^ However, imposing sequence restrictions on supramolecular polymerisation from monomer design – solely based on structural complexity and geometry – for the construction of 2D materials currently seems to be a difficult, yet stimulating challenge for supramolecular chemists.^41^

### 2.4. Structural control

A variety of physicochemical processes can be used to control the polymerisation energy pathway and thus select a potential 2D supramolecular structure:

#### Crystallisation-driven self-assembly

The strategy involves using crystalline seeds to initiate the elongation of supramolecular 2D materials.^42,43^ By combination of seeds and monomer design, the aspect ratio of 2D supramolecular systems can be modulated from rectangular,^44,45^ to spear-like,^46^ hexagonal,^47^ or diamond shapes.^48^ Crystallisation-driven assembly shows interesting properties towards multidimensional control: (i) sequential addition of different monomers allows assembly of 2D structures with different block compositions and functionalisation areas; (ii) the crystalline seed at the core of these nanosheets can be selectively dissolved to create hollow 2D assemblies;^44^ (iii) external stimuli (*e.g.* temperature) can promote size-controlled 1D to 2D transitions.^49^

#### Reaction-driven self-assembly

Dormant monomers can be chemically activated into self-assembling units that spontaneously undergo supramolecular polymerisation.^50,51^ Dynamic changes in the molecular structure can trigger controlled assembly pathways, for example by connection of aliphatic tails and hydrophilic units into self-assembling amphiphiles for 1D^52^ and 2D elongation.^53,54^

#### Frustrated assemblies

As proven by a series of studies, the dynamic nature of certain supramolecular assemblies can result in the creation of structural defects.^55^ Even minor conformational distortions can lead to significant deformations in the final assemblies that hamper supramolecular propagation. Understanding this growth frustration by monomer design could be one of the most challenging, yet probably also most rewarding strategies, to control the size and shape in 2D supramolecular systems.^56^ In certain cases, implementation of comonomers with geometrical restrictions can be used to control the structural fate of the final assemblies.^57^ Frustration of the crystallisation process can also be achieved by monomer composition to obtain complex structures such as toroidal micelles with a tuneable size.^58^

#### Stoppers

Another approach to control the dimensions of supramolecular assemblies is the use of one-faced monomers that work as stoppers, preventing supramolecular propagation in a specific dimension.^59^ Aida used this approximation to cap the growth of 1D supramolecular protein polymers.^60^ Recently, the use of a reductant-sensitive molecular stopper was reported to control on demand the size of supramolecular polymers.^61^ Implementation of stoppers to control 2D supramolecular growth would require the precise adjustment of structure dynamics and monomer exchange to allow the efficient and selective capping of growing polymer ends.

Withal, supramolecular chemists aspire to master all the mentioned factors to finely control the fate of the polymer's morphology and potential responsiveness. However, the molecular complexity required to control the dynamic nature of non-covalent bonds by design could be very high, which challenges the synthetic chemist in exploring new monomer possibilities.

## 3. Directing monomer self-assembly in 2D

**Fig. 2:**
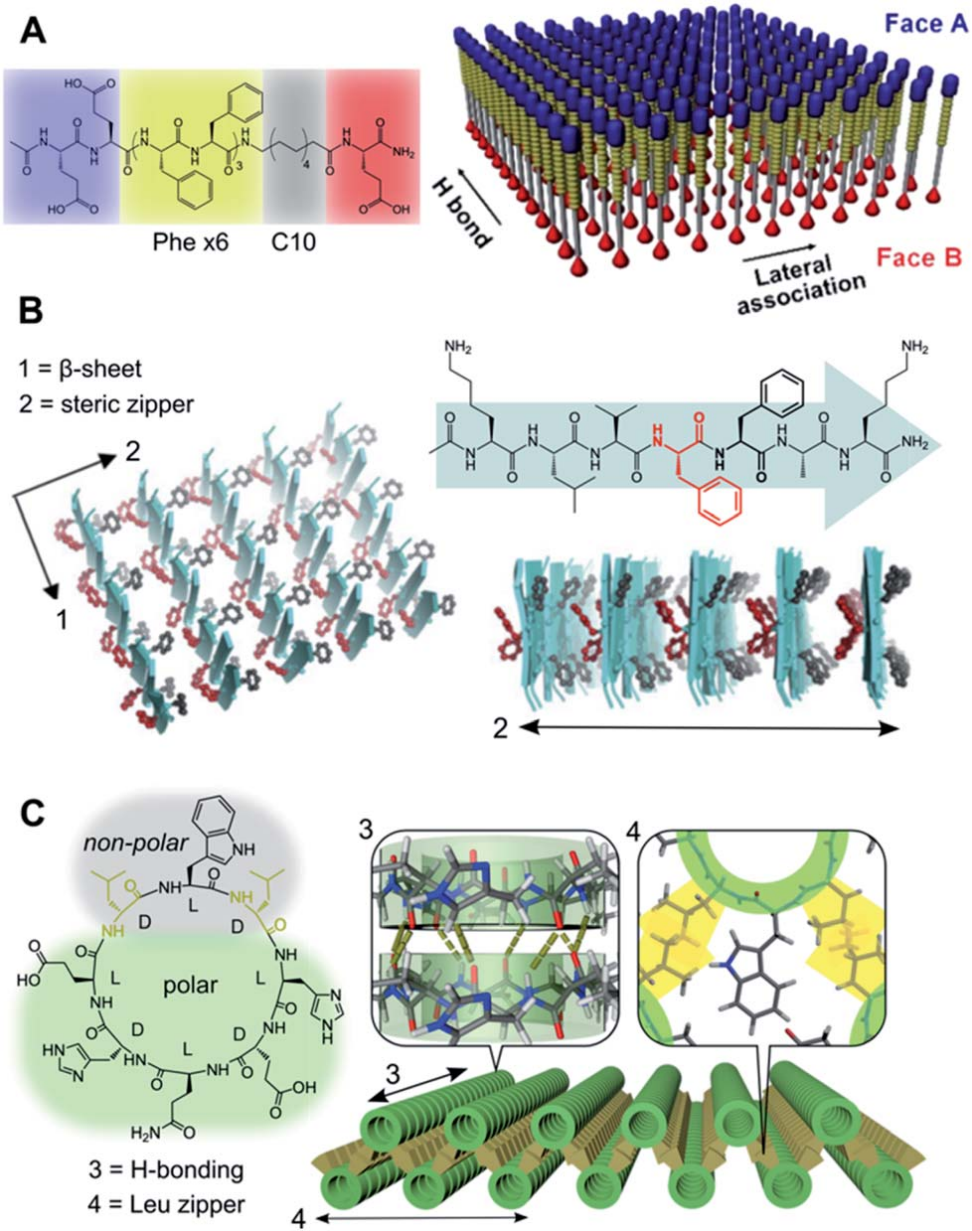
Hydrophobic packing of triblock (A and B) and diblock (C) peptide amphiphiles as mono- and bilayers, respectively. (A) Janus nanosheets obtained from peptides with segregated hydrophobic blocks (Phe_6_*vs.* C10), which favour parallel monomer alignment by Phe H-bonding and π–π stacking in two perpendicular axes. Reprinted with permission from ref. 70. Copyright 2017 American Chemical Society. (B) Steric zipping of complementary hydrophobic interfaces between adjacent β-sheets. Reprinted with permission from ref. 71. (C) Amphiphilic cyclic peptide with alternating (d/l) chirality with 2D self-assembly *via* inter-backbone axial H-bonding (3) and lateral Leu zippers between nanotubes (4). Adapted with permission from ref. 65. Copyright 2019 American Chemical Society.

It is key to note the importance of directionality in non-covalent interactions to ensure *structural order* and *pathway simplicity* in 2D self-assembly. For example, directional interactions such as H-bonding and π–π stacking can guide the ordered elongation of monomers across multiple dimensions (Fig. 1).^62^ However, non-directional forces (*e.g.* hydrophobic effects) are valuable orthogonal motifs that require the assistance of directional interactions to facilitate supramolecular polymerisation in 2D. Pre-folded scaffolds, such as α-helical peptide backbones^63,64^ or self-assembled nanotubes,^65^ can also direct monomer elongation by segregating supramolecular motifs into domains with defined geometries (*e.g.* Leu zippers (Fig. 2C) or polar contacts (Fig. 4B)). With these ideas in mind, four important design concepts that provide directionality and internal order to the assembly of 2D supramolecular systems will be discussed in this section.

### 3.1. Engineering directionality in hydrophobic packing

Ubiquitous for amphiphilic monomers in water, the hydrophobic effect constitutes a primary driving force in aqueous self-assembly.^66,67^ A variety of amphiphilic monomers have been assembled in water across two dimensions, all of which capitalise on burying different hydrophobic segments in the core of a supramolecular ensemble. Alternatively, liquid–liquid interfaces can template 2D self-assembly based on the partial solubility of different monomer domains.^68^ Structure-wise, these amphiphilic monomers can be split into two broad categories: diblock and triblock amphiphiles (Fig. 2). Whereas diblock monomers lead to bilayers, triblock amphiphiles present a polar–hydrophobic–polar structure that allows the assembly of monolayers.^69^ Importantly, triblock monomers can be arranged in parallel or antiparallel orientation based on the directionality of their non-covalent binding domains. Stevens *et al.* developed parallel triblock amphiphilic assemblies by implementing mixed aliphatic and Phe cores, so H-bonding and π–π stacking between Phe units drive face separation into Janus nanosheets (Fig. 2A).^70^ Alternatively, an antiparallel order was achieved by hydrophobic ‘steric zippers’ in cationic triblock amyloid-forming peptides, where the antiparallel β-sheet stacking of monomers creates complementary open/closed Phe interfaces (Fig. 2B).^71^ It should be noted that the presence of ionic terminal moieties normally requires the adjustment of pH and/or ionic strength to externally control 2D self-assembly. These examples illustrate the two perpendicular axes of growth of peptides in the β-sheet configuration: backbone H-bonding *versus* side-chain interactions,^72^ directing the monomer orientation in a 2D lattice from the hydrophobic block (Fig. 2). Beyond peptide monomers, triblock bolaamphiphiles capitalise on the incorporation of mesogenic core units (*e.g.* biphenyl^73^ or pyrene^74^) and hydrophilic/ionic termini that prearrange their hydrophobic packing.

In contrast to triblock monomers, diblock amphiphiles can self-organise into 2D bilayers by both lateral and axial hydrophobic packing. Rigid diblock monomers can also incorporate ionic domains to control directional growth, which allows the ordered assembly of 2D bilayers from flat amphiphilic peptoids^75^ and cylindrical coiled-coils.^57^ Our group recently reported cyclic d/l-alternating peptides that grow 1D nanotubes *via* backbone H-bonding,^76^ whose hydrophobic face induces their subsequent assembly into 2D bilayers by inter-tubular hydrophobic interactions (Fig. 2C).^65^ In this design, interlayer Leu–Leu contacts at specific angles afforded staggered nanotubular bilayers, providing an excellent monomer scaffold for further functionalisation in all three dimensions.^77,78^

The examples in Fig. 2 showcase the ability of directional interactions, such as H-bonding, and nanotube interfaces to guide the hydrophobic packing of monomers, which becomes evident in the perfect alignment of monomers throughout the entire internal structure of these 2D assemblies.

### 3.2. π–π stacking of rigid molecular blocks

**Fig. 3:**
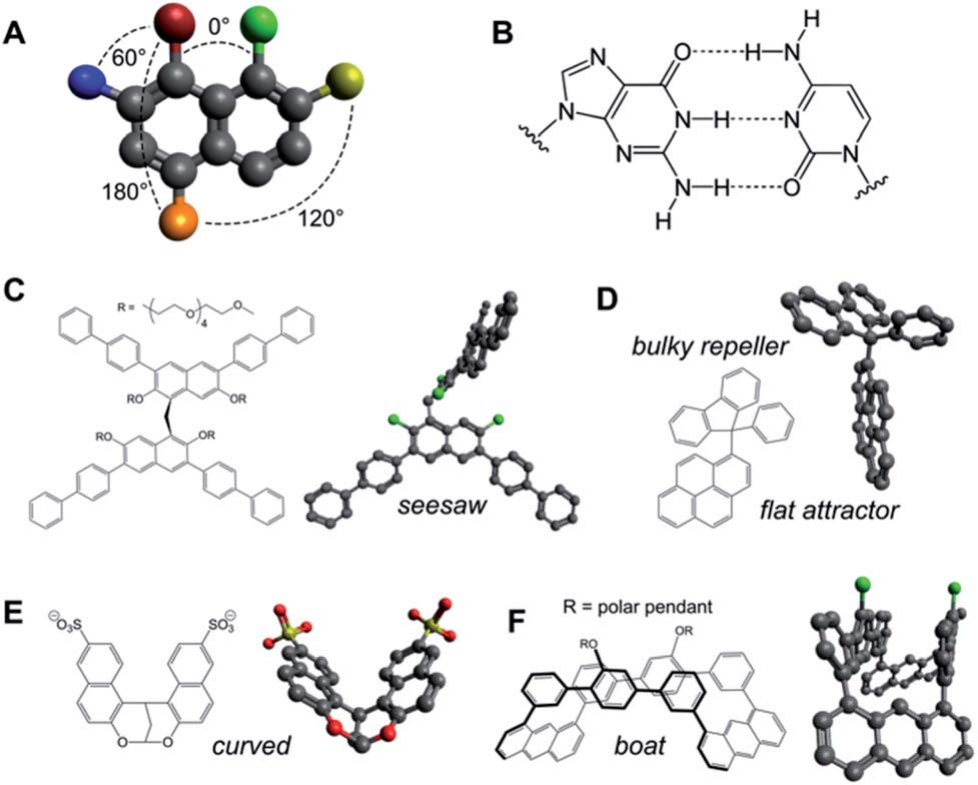
(A and B) Aromatic scaffolds allow precise control over the geometrical disposition of their substituents (A) and the establishment of complementary multivalent interfaces (*e.g.* nucleobases, B). (C–F) Examples of rigid aromatic monomers capable of 2D self-assembly and their minimised 3D structures (Avogadro v.1.2.0): seesaw (C),^80^ attractor–repeller (D),^81^ curved (E)^82^ and boat-shaped (F);^83^ green substituents represent polar pendants.

Aromatic rings and polycycles have been widely explored in 2D self-assembly capitalising on three particular features: (a) the oriented disposition of substituents at specific angles in the same plane (*e.g. n*·60° in benzene derivatives; Fig. 3A and B); (b) the rigidity of aromatic units allows the geometrical design of polycyclic shapes and interfaces for complementary stacking in a variety of 2D arrays; and (c) the directional interaction between π electron clouds (*δ*−) and electron-deficient atoms (*δ*+) directs their hydrophobic packing in a dipole–dipole fashion. Aromatic geometry can range from simple flat pyrene interfaces,^26^ to perpendicular aromatic diblocks,^79^ seesaw-shaped scaffolds,^80^ dual planar ‘attractor’ and non-planar ‘repeller’ surfaces,^81^ curved monomers^82^ and boat-shaped building blocks,^83^ amongst others (Fig. 3C–F).

Porous nanosheets have been also assembled from aromatic macrocycles using either covalent macrocyclic tiles of fixed geometry (Fig. 5A)^14^ or fully supramolecular tiles based on the cation–π stacking of flat aromatic monomers.^84^ Overall, these designs demonstrate the importance of the geometrical precision at which aromatic interfaces can be synthesised, posing the challenge of incorporating more complex aromatic monomers such as helicenes^85^ and porous aromatic frameworks^86^ into 2D self-assemblies.

Aromatic scaffolds also serve as rigid units to segregate polar and hydrophobic substituents at specific angles, allowing the 2D self-assembly of diblock^87^ and triblock^88^ amphiphiles (Section 3.1) that capitalise on π–π interactions to order their hydrophobic cores. Importantly, variations in the pendant structure allow the tuning of the nanosheet aspect ratio.^89^ Connection of aromatic cores to stimuli-responsive polar pendants has also been recently employed for the assembly of reversible aqueous supramolecular polymers.^80,83^ Similar to supramolecular assemblies, dynamic covalent chemistry offers alternative access to highly crystalline 2D networks from aromatic monomers with potential structural reconfiguration through covalent bond exchange.^90^

### 3.3. Host–guest chemistry

**Fig. 4:**
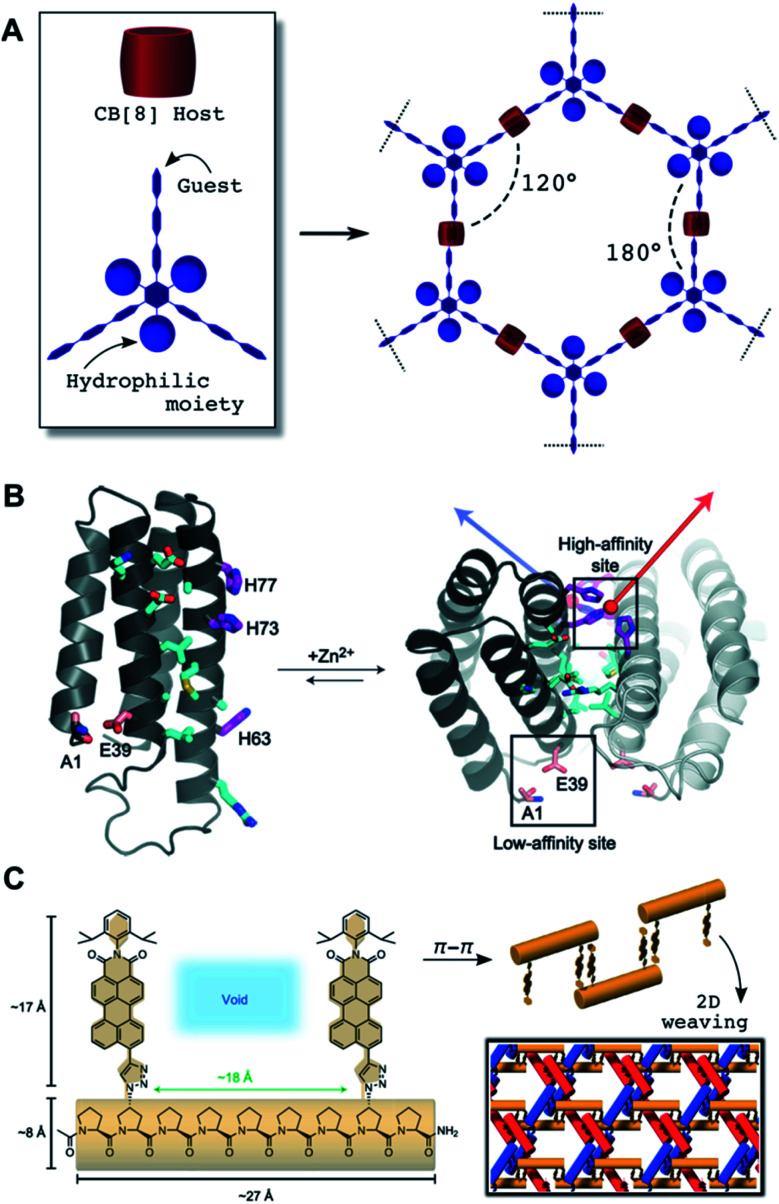
(A) Illustration of geometric control attainable from trigonal multivalent host monomers and cucurbituril CB[8] cavitands as porous hexagonal 2D networks. (B) 2D self-assembly topologically encoded on the outer surface of pre-folded protein four-helix bundles: Two high-affinity Zn^2+^ coordination sites allow for the elongation of this protein in helical 1D assemblies, while a low affinity site provides a perpendicular elongation point for 2D growth. Polar contacts at interfacing protein domains (cyan) contribute to the stabilisation of the assembly. Reprinted with permission from ref. 101. Copyright 2012 Springer Nature. (C) Woven 2D assembly obtained from oligoproline monomers elongated though π–π stacking of their aromatic pendants. Each individual 1D suprapolymer (red, blue and orange) interlocks by top-down alternation of monomers, thus leaving periodically spaced voids filled by the other 1D chains. Adapted with permission from ref. 123. Copyright 2017 Springer Nature.

Supramolecular host–guest interactions involve the binding between converging (host) and diverging (guest) binding sites based on non-covalent interactions and hydrophobic effects.^91^ Integration of host–guest pairs as ‘mortar’ to specifically bind cross-linking ‘bricks’ has been widely exploited to build 2D supramolecular networks.^92^ A structural requirement for the host–guest complex is to preserve the molecular directionality of the building blocks to prevent cross-linking in the perpendicular axis of the nanosheet. A variety of cavitands have been explored in the assembly of supramolecular 2D nanostructures, as reviewed in the literature.^93^ Inspection of the reported systems reveals a few recurrent structural features. One is the use of planar building blocks, which in some cases introduce π–π stacking as the driving force into the formation of the host–guest complex within the cavitand. Additionally, the inclusion of moieties that prevent 3D growth is frequently required. A remarkable example combining both of these features is the formation of 2D honeycombs with CB[8]-based supramolecular complexes.^94^ The overall assembly is defined by a multivalent guest scaffold and its cross-linking with CB[8] units (Fig. 4A). Importantly, suppression of interlayer stacking can be achieved by incorporation of hydrophilic pendants into the planar scaffold. Refinement of this architectural concept aimed to release molecular strain to yield larger nanosheets,^95^ provide stimuli-sensitivity^96^ and attach specific fluorophores.^97^ The use of semiflexible chains resulted in the generation of polymorphic 2D structures with lyotropic properties.^98^ In addition to cucurbituril cavitands, some other host scaffolds such as calixarenes and cyclodextrins have been integrated into 2D self-assemblies.^93^ Lacking the capabilities of forming ternary host–guest systems, these supramolecular connectors are less predictable in their final orientation geometry. Therefore, 2D host–guest designs must apply certain strategies to direct their planar elongation, such as amphiphilic coronas to prevent the stacking of the aromatic guest,^99^ or the inclusion of multivalent synthetic hosts/guests that control the cross-linking and branching of the supramolecular ensemble.^100^

### 3.4. 2D macromolecular assemblies

Macromolecules can act as monomers by exploiting their pre-folding into rigid scaffolds to orient multivalent domains for 2D self-assembly. Biomacromolecules provide inspiration for synthetic designs based on their intrinsic ability to adopt rigid backbone folds (*e.g.* α-helices and duplexed DNA). For example, helical peptide and protein scaffolds have been assembled in 2D by controlling binding domains such as metal coordination groups (Fig. 4B),^101^ oppositely charged amphiphiles,^102^ hydrophobic zipping of coiled-coils^64^ and trigonal orientation of orthogonal domains.^63^ Protein interfaces have also produced porous 2D assemblies by geometrically restricted oligomerisation of covalent protein dimers^103^ and hydrophobic interfaces.^104^ Even allosteric control has been introduced in protein blocks for supramolecular 1D–2D switching.^105^ DNA nanotechnology has provided unparalleled design flexibility to access increasingly complex 2D suprastructures, from patterned DNA origami wireframes,^106,107^ to honeycomb DNA networks,^108,109^ DNA tile arrays^110,111^ and hybrid DNA–polymer nanoassemblies,^112^ amongst others.^113^ Thus, DNA hybridisation capitalises on directional inter-strand H-bonding and intra-strand π–π stacking between nucleobases^114^ to assemble helical blocks into a myriad of crystalline materials.^115^

Unlike proteins and nucleic acids, most non-biogenic polymers are not folded into precise secondary structures, hence requiring alternative mechanisms to achieve 2D growth. Crystallisable blocks have been used to provide internal order to the hydrophobic core of the assembly.^116–118^ An important breakthrough was made by developing crystallisation-driven self-assembly methodologies (Section 2.4).^119^ Note that many of these examples use organic (co)solvents for self-assembly, posing the standing challenge of translating these supramolecular concepts into physiological environments. Woven 2D assemblies have been recently developed by metal coordination of polycyclic monomers into pre-assembled tiles for intertwined 1D covalent polymerisation.^120,121^ Other metal–organic suprapolymers can result in dynamic 2D assemblies, from woven to ordered complexes and randomly entangled networks.^122^ Oligoproline monomers provide a metal-free and fully supramolecular alternative for 2D weaving based on π–π stacking of aromatic pendants at triaxial crossing points (Fig. 4C).^123^ Woven 2D nanomaterials demonstrate the structural complexity that can be engineered at the molecular level from suitable monomers equipped with directional binding motifs, providing valuable inspiration for new designs to come.

## 4. Edge applications

Supramolecular materials can bring advantages and new properties such as fine tuning of chemical topology and shape, adaptation to their environment and reversible structural transitions. Capitalising on these interesting properties, a wide variety of applications have already been proposed for 2D supramolecular systems.^124,125^ A short collection of recent applications will be briefly discussed below to highlight the strong potential of 2D assemblies and the clear necessity of new monomers and conceptual designs.

### 4.1. Chemical separation

**Fig. 5:**
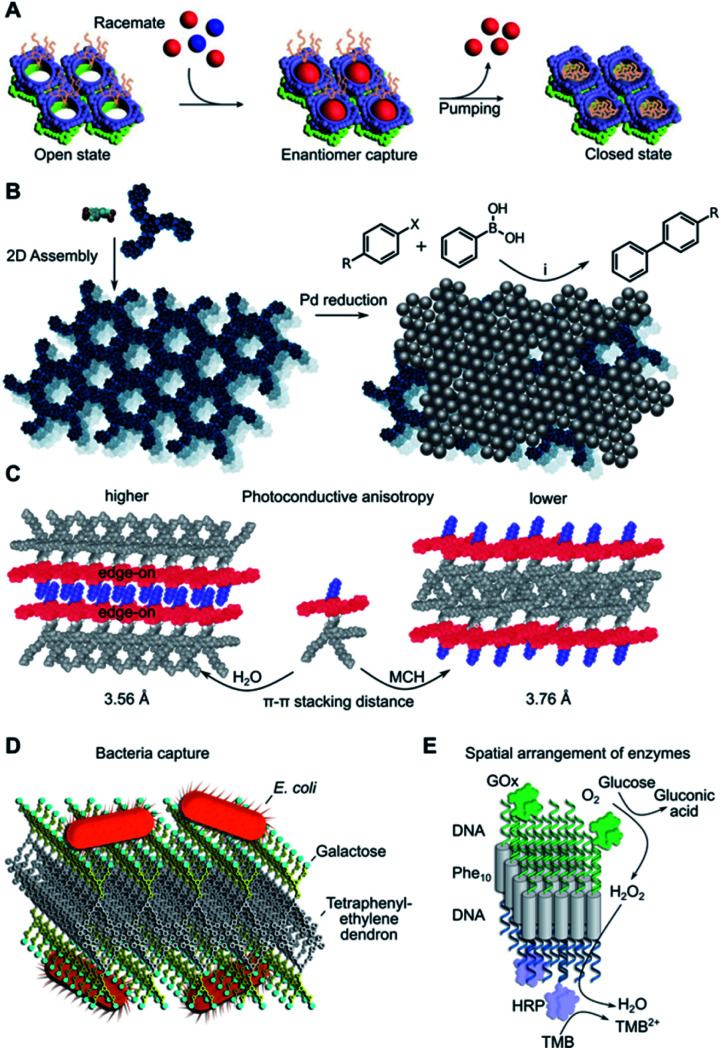
Edge applications of supramolecular 2D assemblies. (A) Enantiomeric molecular recognition and pumping of racemic guests on chiral 2D porous nanosheets. Adapted with permission from ref. 14. Copyright 2018 Springer Nature. (B) Immobilisation of Pd nanoparticles on 2D nanosheets and subsequent catalytic Suzuki–Miyaura coupling. X = I or Br, R = H, CH_3_, CH_3_O, NO_2_, CHO or CN, (i) K_2_CO_3_, ethanol, 65 °C. (C) Solvent-dependent self-assembly results in different supramolecular π-stacking and photoconductive anisotropy. MCH = methylcyclohexane. (D) Bacterial agglutination mediated by galactose-decorated nanosheets. Adapted from ref. 143. Copyright 2020 Wiley-VCH. (E) DNA-functionalised enzymes anchored selectively to each side of a DNA–peptide–DNA hybrid Janus nanosheet. GOx = glucose oxidase; HRP = horseradish peroxidase; TMB = 3,3′,5,5′-tetramethylbenzidine. Adapted from ref. 149. Copyright 2021 Wiley-VCH.

2D nanosheets have potential use in several separation problems due to the occurrence of nanopores in these materials. A particularly remarkable example was reported by Sun *et al.* in the separation of racemic mixtures of l and d-tryptophan by macrocycle-based homochiral porous nanosheets (Fig. 5A).^14^ This system can close the pores *on-demand* by addition of ammonium acetate, thus, the selective binding of the d-stereoisomer can be prevented or expelled from the 2D matrix if already captured. The size of these pores can be adjusted at the monomer synthesis stage, suggesting the possibility of adapting this design for the stereo-selective capture of other molecular targets. Some other examples in this sense have been reported, with excellent enantiomeric separation (>99% ee).^126^ Additionally, highly porous 2D assemblies can be used for the size-selective filtration of nanoparticles,^96,127^ where the thermal expansion of the pores can tune the size separation cut-off according to the pore distribution achieved.^96^

### 4.2. Catalysis

Porous 2D nanosheets can incorporate a range of catalytically active nanoparticles and thus prevent their aggregation-induced loss in catalytic activity. Some examples include the catalysis of the Suzuki–Miyaura coupling reaction on the surface of PdNPs^84^ (Fig. 5B) or electrochemical nitrogen reduction mediated by PtNPs.^128^ A selective approach can take advantage of precisely defined molecular-sized pores to allow specific molecular transformations within nanosheet environments. This strategy must consider the molecular dimensions and chemical properties of the chosen reactant. Some illustrative examples include the macrocyclisation of linear pre-organised substrates within the pores of carefully designed 2D nanosheets *via* Suzuki–Miyaura coupling,^129^ or in-pore asymmetric catalysis of small substrates based on their enantio-specific fixation.^130^ Questions arise on the catalytic limitations of these nanopores in comparison to enzymatic binding pockets, where active groups are precisely positioned to manipulate the substrate conformation and its catalytic transformation.^131,132^

### 4.3. Charge transport

Supramolecular chemists have also designed bottom-up 2D materials with optical and electronic properties. The precise orientation and spacing of monomers in the assembled state provide an opportunity to modulate charge transport processes. While this phenomenon is well known in organic semiconductors,^133^ less attention has been focused on 2D nanosheets. New developments in this field include the assembly of aromatic amphiphiles as 2D nanosheets with high anisotropic charge-carrier mobility, which results from the defined orientation of monomers in the 2D lattice (Fig. 5C).^134,135^ Other molecular designs have shown similar properties,^88,136^ supporting the correlation between precise monomer packing parameters and the specific charge transport capabilities of the material. However, it is still unclear whether proton transport could benefit from a similar lattice effect in 2D systems.^137,138^ Future efforts to measure and understand charge transport processes in new 2D supramolecular materials are necessary for their implementation in next-generation fuel cell membranes, photovoltaic devices and sensors.

### 4.4. Biological applications

2D assemblies are present in many biological structures, so it is not surprising that many supramolecular nanosheets are suitable materials for biological and biomedical applications.^8,139^ High surface multivalency in 2D is useful for the development of sensitive biosensors,^15,16,140,141^ or to capture microorganisms such as bacteria^142,143^ or viruses (Fig. 5D).^71^ The stimuli-responsiveness of some nanosheets can be used for the controlled release of drugs, such as light-triggered doxorubicin release,^144^ or as bacteria sensors with fluorescence output after enzymatic degradation of the assembly.^145^ Incorporation of fluorophores into the nanosheets opens access to photodynamic therapy applications^146^ and sensitive biocompatible probes.^147,148^ Fine control over monomer packing allows for the assembly of Janus nanosheets with distinct enzymatic activities on either leaflet (Fig. 5E).^149^ Other structures may be of interest for future tissue regeneration applications, such as 2D sheets that serve as templates for mineralisation by calcium carbonate deposition,^150^ or dynamic nanosheets with self-healing properties.^12,151^ Even though this is still a nascent field, these examples highlight the vast potential of supramolecular nanosheets as advanced responsive materials in biology and biomedicine.

## 5. Outlook

New supramolecular strategies are emerging for the assembly of two-dimensional materials that could combine molecular order, function and adaptive behaviour. These bottom-up approaches improve the synthetic access to 2D materials, as well as surmount the additional heterogeneous modification steps that could be required in classical top-down strategies. In this perspective, we have highlighted selected examples of current strategies and potential future challenges in the assembly of 2D supramolecular materials. From the design standpoint, combining orthogonal supramolecular interactions and preventing layer stacking have been proved critical to obtaining controlled bidimensional growth. A careful selection of monomer geometry and directional non-covalent forces is needed to obtain smooth free-energy pathways that will lead to discrete 2D nanostructures. Regarding reversibility, multi-monomer systems should avoid potential kinetic traps to allow monomer exchange and structural reshuffle along the whole assembly pathway. Sensitivity to dilution could result in different degrees of polymerisation, which may require post-assembly cross-linking at the expense of potential dynamic network reconfiguration. Beyond stimuli responsive behaviour, the potential self-assembly of multicomponent 2D functional systems, with a monoatomic thickness, also remains elusive. Overall, the successful development of effective 2D nanomaterials and their future impact as a reliable technology will depend on efficient and predictable strategies at the lowest possible synthetic cost. Here, the *supramolecular approach* is yet to show its real potential to achieve control of properties and functions, which will not be accessible by static covalent strategies. The development of new monomers and the accurate understanding and prediction of their hierarchical 2D self-assembly at the molecular level, constitute a fundamental, yet not trivial exercise in this research field. The growing number of different chemical concepts and structural designs applied to the assembly of 2D supramolecular systems indicates that an important effort is still needed and currently ongoing in these directions.

## Note from the authors

In the interest of conciseness, the examples included in this perspective have been limited to a representative collection of published structures and mechanistic studies. The authors apologise for any insightful reports that might have been left out in this manuscriptt.

## Author contributions

I. I. drafted the first version of the manuscript with contributions from all authors. All authors reviewed and contributed to the final version of the paper.

## Conflicts of interest

There are no conflicts to declare.

## Supplementary Material
